# Associations between transition to retirement and changes in dietary intakes in French adults (NutriNet-Santé cohort study)

**DOI:** 10.1186/s12966-017-0527-6

**Published:** 2017-05-30

**Authors:** Wendy Si Hassen, Katia Castetbon, Eva Lelièvre, Aurélie Lampuré, Serge Hercberg, Caroline Méjean

**Affiliations:** 1 Université Paris 13, Sorbonne Paris Cité, Centre de Recherche en Epidémiologie et Statistique, Equipe de Recherche en Epidémiologie Nutritionnelle EREN, Inserm (U1153), Inra (U1125), Cnam, Université Paris 13, F-93017 Bobigny, France; 2 Université Libre de Bruxelles, Ecole de Santé Publique, Centre de recherche en Epidémiologie, Biostatistiques et Recherche Clinique, Route de Lennik 808 – CP 598, 1070 Brussels, Belgium; 3 Institut National des Etudes Démographiques, 133 boulevard Davout, 75020 Paris, France; 4 Département de Santé Publique, Hôpital Avicenne, F-93000 Bobigny, France; 5 Insititut National de la Recherche Agronomique INRA, UMR 1110 MOISA, F-34000 Montpellier, France

**Keywords:** Dietary intake, Life course, Prospective study, Retirement

## Abstract

**Background:**

Few studies have focused on the influence of retirement on dietary behaviors. Our study aimed at assessing the associations between transition to retirement and changes in dietary intake in French adults, particularly according to spousal retirement and baseline income.

**Methods:**

This prospective study included 577 French participants from the NutriNet-Santé cohort who retired over a 5-year follow-up (2009–2014 or 2010–2015). At baseline and every year, dietary intakes were assessed using 24 h records. Repeated measures of dietary intake were analysed using mixed models adjusted for energy with random effects of time and period (before and after retirement) to assess changes following retirement for each gender.

**Results:**

After retirement, intakes of saturated fatty acids and sodium increased in both genders. Women showed specific changes after retirement: decrease in the score of adherence to recommendations and in intakes of fruits, proteins, vitamins; increase in intakes of fatty sweet products. In men with the lowest income at baseline, specific changes in intake were associated with retirement such as decrease in intake of dairy products and increase in intake of lipids.

**Conclusions:**

Transition to retirement was associated with unhealthier dietary intakes. These results may help defining interventions during this vulnerable life-period.

**Trial registration:**

This study was conducted according to guidelines laid down in the Declaration of Helsinki and all procedures were approved by the Institutional Review Board of the French Institute for Health and Medical Research (IRB Inserm No. 0000388FWA00005831) and the French Data Protection Authority (Commission Nationale Informatique et Libertés No. 908450 and No. 909216). Electronic informed consents were obtained from all participants.

**Electronic supplementary material:**

The online version of this article (doi:10.1186/s12966-017-0527-6) contains supplementary material, which is available to authorized users.

## Background

Numerous clinical and epidemiological studies have highlighted the important role of diet as a risk or a protective factor of chronic diseases, such as cardiovascular diseases, some cancers, type 2 diabetes, and hypertension, for which incidence substantially increases from mid-age [1, 2]. Life course approach is increasingly developed in chronic disease epidemiology while the influence of life course on dietary behaviors has been little studied so far [3, 4]. The available studies on the impact of the life course on diet have mostly highlighted the contribution of biological-based aspects of the life course on the development of chronic diseases [3, 5, 6]. However, some studies have focused on the contribution of the life course approach for identifying social and individual determinants of dietary behaviors during life [4, 7].

Retirement appears to be a major transition in the life course which may affect lifestyle components, such as dietary patterns, physical activity, smoking or alcohol consumption [8–12]. To our knowledge, among available studies on the relationships between retirement and diet, nine used a prospective design which allowed assessing the associations between transition to retirement and dietary intake over time [8]. Most of these focused on specific food groups such as fruit and vegetables or specific nutrients such as energy, protein or fibre, and did not consider overall diet [8]. A recent review showed that these prospective studies reported mixed impact of retirement on dietary intake and dietary behaviors and has shown equivocal results [8]. Such heterogeneity could be explained by differences in length of follow-up and consequently, persistence of the effects of retirement on diet, and in study design. In addition, important differences between state retirement policies (legal age to retirement, retirement contributions and pensions, etc.) may also explain such equivocal results. Two prospective studies showed a positive impact of retirement on dietary intake: higher adherence to several Nordic recommendations were observed in women who retired compared with continuously employed women, and increase of vegetables after retirement in men was reported in another study [13, 14]. However, results on food groups’ consumption are equivocal [15–17]. Regarding nutrient intake, findings are not concordant [8]: either decrease, no difference or increase in the considered nutrient intakes were observed in previous studies [15–18].

To our knowledge, associations between retirement and the different aspects of diet all together (overall quality score, food groups and nutrients) have not been assessed and socio-demographic or economic characteristics of subjects have not been taken into account. As retirement has been described to be associated with a decrease in income and decrease in expenditure, transition to retirement may affect differently dietary behaviors of individuals according to their level of income before retirement [19, 20]. Spousal status and retirement have been mentioned as factors influencing food expenditure but influence of retirement of the spouse on intake needs further investigation [21, 22]. Therefore the aim of our study was to assess the impact of transition to retirement on food and nutrient intakes as well as adherence to nutritional guidelines using repeated data in French adults over a 5-year follow-up. In addition, we assessed differences in the association between transition to retirement and dietary intake according to spousal retirement status and baseline household income.

## Methods

### Subjects

Subjects were adults participating in the NutriNet-Santé study, a large web-based prospective observational cohort launched in France in 2009, implemented in the general population. The design, methods and rationale have been described previously [23]. Briefly, participants were included in the cohort once they completed a baseline set of questionnaires assessing dietary intake, physical activity, and socioeconomic and health status. As part of their follow-up, the participants completed the same set of questionnaires every year.

The present analysis focused on participants included in the NutriNet-Santé cohort study between May 2009 and April 2010, aged between 50 and 64y, who were working at baseline, and retired during follow-up.

### Ethics, consent and permissions

This study was conducted according to guidelines laid down in the Declaration of Helsinki and all procedures were approved by the Institutional Review Board of the French Institute for Health and Medical Research (IRB Inserm No. 0000388FWA00005831) and the French Data Protection Authority (Commission Nationale Informatique et Libertés No. 908450 and No. 909216). Electronic informed consents were obtained from all participants.

### Assessment of dietary intake

At baseline and every year during follow-up, participants were invited to fill in three validated self-administered web-based 24-h dietary records, randomly assigned over a 2-week period (2 weekdays and 1 weekend day) [23–26]. The web-based dietary assessment method relies on a meal-based approach, recording all foods and beverages (type and quantity) consumed at all eating occasions during the day. Participants estimated portion sizes according to standard measurements or using validated photographs [27]. The values for energy, macronutrients and micronutrients were estimated using published nutrient databases [28]. Energy-under-reporting participants were identified and excluded using the method proposed by Black [29]. From 24-h dietary records, the French Programme National Nutrition Santé-Guideline Score (PNNS-GS), reflecting adherence to nutritional recommendations was computed each year of the follow-up.

The 15-point PNNS-GS is a validated a priori score, which has been extensively described elsewhere [30]. Briefly, it includes 13 components: eight refer to food-serving recommendations (fruit and vegetables; starchy foods; whole grain products, milk and dairy products; meat, poultry, eggs; fish and seafood; vegetable fat; water vs soda), four refer to moderation in consumption (added fat; salt; sweets; alcohol) and one component pertains to physical activity [30]. For the analysis, we consider a modified version of the PNNS-GS, the m-PNNS-GS which takes into account only the dietary components, excluding the physical activity component. The mPNNS-GS has a maximum of 13.5 points; a high score reflects behavior which is in accordance with national recommendations.

### Sociodemographic and socioeconomic indicators

At baseline and each year at follow-up, socioeconomic and demographic data were collected using a web-based self-reported questionnaire [31]. Employment status was coded into eight categories: professionally active individuals, unemployed persons, never employed individuals, retired, students, homemakers, disabled, vocational trainees. Participants were asked about monthly household income including total salary, social benefits, family allowance, and rental income. Subjects could also choose not to indicate their household income. The monthly household income was calculated according to household composition, reported by the participant. Thus, the reported monthly household income was divided by the number of household units (HU) (1 HU for the first adult in the household, 0.5 HU for other persons aged ≥14 years and 0.3 HU for children <14 years [32]. The following four categories of monthly income were used: <1800€, 1800–2700€, >2700€ per HU, and a category grouping individuals who chose not to indicate their household income. Thanks to a web-based questionnaire, participants were asked to specify their marital status and the employment status of their spouse, which was coded similarly as described above.

### Statistical analyses

The present analysis focused on participants living in mainland France, who did not under-report energy intake, and who had at baseline (2009 or 2010) and 5 years later (2014 or 2015, end of the follow-up) at least three completed 24-h dietary records and no missing data for socioeconomic and demographic characteristics. Database was constituted of repeated yearly measures for dietary intake and socioeconomic indicators in each participant. A set of 24-h dietary records could therefore be collected every year, leading to a maximum of 5 collected sets of 24-h dietary records per subject. Retirement event was determined by comparing the employment status at year *n-1* and year *n*. We defined a period indicator according to the self-reported year of retirement: the period before retirement corresponded to the period from baseline to the year of retirement (included), and the period after retirement was the period with data collected after the year of retirement.

Comparisons of socioeconomic and demograhic characteristics and dietary intakes at baseline between men and women were performed using Student’s t-test and chi-square test, as appropriate. The repeated measures of dietary intakes for each participant were analysed using mixed models adjusted for total energy intake with random effects of the time and the period (before and after retirement) and random intercept to assess changes following retirement for each gender.

Both time scale and the period indicator (before and after retirement) were included in the models in order to evaluate the association between transition to retirement and dietary intakes, independently of changes over time. Analyses were performed separately in men and women as it has been suggested that some differences based on gender existed through retirement [8, 21]. Changes in energy, nutrients, and food groups’ intake, as well as adherence to French dietary guidelines through the mPNNS-GS score were studied.

Spousal retirement was also determined by comparing spousal employment status at baseline and at the end of the follow-up. When interactions were significant (*p*-value < 0.2), stratified mixed models according to spousal retirement status and baseline household income were performed to assess the differences in the association between transition to retirement and dietary intake. In order to take into account multiple comparisons, significance level was set at a *p*-value <0.01. Data management and statistical analyses were performed using SAS (version 9.3; SAS Institute, Inc., Cary, NC, USA).

## Results

### Description of the sample

Among 12,549 participants included between May 2009 and April 2010 without missing data for sociodemographic or economic and dietary factors at baseline and at the end of the follow-up, 5342 individuals (42.6%) were aged between 50 and 64 years, 1001 retired during follow-up (8%). Among them, 198 participants were not professionally active at baseline and 226 retired during the last year of follow-up - leaving 577 individuals (402 women and 175 men) for analysis. In our sample, 89% of individuals had at least 4 yearly sets of 24-h dietary records. Percentages of subjects <60 years, employees and intermediate professions, individuals with undergraduate educational level, those belonging to the intermediate income class were higher in women compared with men while percentages of managerial staff, manual worker and self-employed, subjects with post-graduate education and individuals in the highest income class were lower (Table 1). At baseline, men had higher intakes of red and processed meats, cheese, alcoholic beverages and total energy (Table 2).

**Table 1:** Socioeconomic and demograhic characteristics of the sample at baseline (*N* = 577)

	Individuals who were employed at baseline and who retired during the follow up (*N* = 577)		
	Women (*N* = 402) %	Men (*N* = 175) %	*P*-value^a^
Age class			<0.0001
50–54 y	13.7	8.0	
55–59 y	62.2	47.4	
60–64 y	24.1	44.6	
Occupation			<0.0001
Manual worker	0.8	2.3	
Employee	20.7	9.7	
Intermediate profession	38.1	13.1	
Self employed	1.7	6.3	
Managerial staff	38.8	68.6	
Education			<0.001
Unknown	0.5	2.3	
Primary	3.0	2.9	
Secondary	35.1	27.4	
Undergraduate	33.3	23.4	
Postgraduate	28.1	44.0	
Household income per consumption unit			0.01
< 1800 euros	15.7	17.1	
1800–2700 euros	26.9	16.6	
> 2700 euros	49.4	61.7	
Unwilling to declare	8.0	4.6	
Marital status			<0.001
Couple (married, partnership…)	71.4	85.1	
Single, divorced	23.1	12.0	
Widowed	5.5	2.9	
Retirement of the spouse during the follow up	*N* = 287	*N* = 149	0.8
No	64.8	63.8	
Yes	35.2	36.2	

^a^ *P*-value for chi-square or Fischer analyses

**Table 2:** Intake of food groups and nutrients and adherence to nutritional recommendations (mPNNS-GS), at baseline

	Women (*N* = 402)	Men (*N* = 175)	
	Mean (SD)	Mean (SD)	*P*-value^a^
Adherence to nutritional recommendations (mPNNS-GS) ^b^ (range 0–13.5)	8.8 (1.6)	8.2 (1.7)	<0.001
Food groups			
Fruit (g/day)	333.5 (182.9)	325.1 (202.7)	0.6
Vegetable (g/day)	264.9 (118.5)	255.9 (124.6)	0.4
Fish (g/day)	49.9 (49.7)	54.6 (52.5)	0.3
Red meat and poultry (g/day)	66.7 (46.2)	88.4 (56.0)	<0.0001
Processed meat (g/day)	26.7 (26.7)	37.9 (37.4)	<0.001
Cheese (g/day)	36.2 (26.4)	46.8 (33.2)	<0001
Starches (pasta, rice, semolina, cereals, potatoes, tubers, bread) (g/day)	170.1 (90.2)	233.6 (118.9)	<0.0001
Whole-grain products (whole-grain cereals, bread, pasta, rice, flour) (g/day)	39.5 (45.8)	47.9 (65.9)	0.1
Fatty and sweet products (g/day)	74.3 (54.7)	92.5 (63.0)	<0.01
Sweet non-alcoholic beverages (g/day)	17.2 (41.5)	24.5 (61.3)	0.1
Butter, margarine (g/day)	17.4 (15.1)	18.5 (15.1)	0.4
Dairy products (cheese, milk, yogurt) (g/day)	180.9 (157.6)	196.7 (139.2)	0.3
Alcoholic beverages (g/day)	90.5 (110.2)	222.8 (232.0)	<0.0001
Nutrients			
Total energy (kcal/day)	1811.9 (354.3)	2290.6 (487.2)	<0.0001
Proteins (g/day)	78.8 (17.8)	94.7 (20.9)	<0.0001
Total carbohydrates (g/day)	187.1 (45.3)	232.8 (59.1)	<0.0001
Complex carbohydrates (g/day)	94.0 (30.8)	127.7 (39.7)	<0.0001
Simple carbohydrates (g/day)	92.6 (28.3)	104.5 (35.2)	0.0001
Fibre (g/day)	21.6 (6.7)	24.0 (8.0)	<0,001
Lipids (g/day)	76.3 (23.5)	93.3 (29.7)	<0.0001
Polyunsaturated fatty acids (g/day)	11.4 (5.9)	14.5 (8.7)	<0.0001
Monounsaturated fatty acids (g/day)	28.7 (9.8)	34.8 (12.5)	<0.0001
Saturated fatty acids (g/day)	30.4 (11.3)	37.0 (12.3)	<0.0001
Cholesterol (mg/day)	304.9 (125.2)	360.9 (164.2)	<0.0001
Omega 3 (g/day)	1.6 (0.9)	1.8 (1.2)	0.02
Omega 6 (g/day)	9.2 (5.5)	12.0 (7.9)	<0.0001
Calcium (mg/day)	947.3 (286.5)	1034.5 (308.0)	0.001
Magnesium (mg/day)	355.7 (97.9)	409.1 (127.7)	<0.0001
Sodium (g/day)	2545.7 (736.8)	3235.6 (973.3)	<0.0001
Beta carotene (μg/day)	4154.2 (2523.9)	3755.6 (2266.4)	0.1
Folate (μg/day)	372.0 (116.2)	385.4 (132.2)	0.2
Vitamin C (mg/day)	141.9 (122.3)	134.8 (77.9)	0.4

*mPNNS-GS* modified French Programme National Nutrition Santé-Guideline Score, *SD* Standard Deviation

^a^p value for Student test analysis

^b^mPNNS-GS: adherence to nutritional guidelines score, based on 24 h dietary records, range 0–13.5

#### Associations between retirement and dietary intakes

In both genders, transition to retirement was associated with increased in intakes of saturated fatty acids and sodium (Table 3). In women only, the transition to retirement was associated with a decrease of the mPNNS score, in intake of fruits, proteins, folate, and vitamin C and an increase in intake of fatty sweet products (Table 3). In addition to the association between transition to retirement and intakes, time was associated with an increase in intake of fatty sweet products and sodium, and a decrease of the mPNNS score and in intake of folate and vitamin C in women, and with an increase in intakes of saturated fatty acids and sodium in men.

**Table 3:** Associations between changes in dietary intakes and transition to retirement in French women (*N* = 402) and men (*N* = 175) (NutriNet-Santé Study) ^a^

	Women (*N* = 402)		Men (*N* = 175)	
	Beta	99% Confidence Interval	Beta	99% Confidence Interval
Adherence to nutritional recommendations (mPNNS-GS) ^b^ (range 0–13.5)	**−0.4** ^**c**^	**−0.6, −0.3**	−0.2	−0.4, 0.1
Food groups				
Fruit (g/day)	**−19.1** ^**d**^	**−35.5, −2.7**	15.3	−12.8, 43.4
Vegetable (g/day)	−5.8	−17.6, 6.0	−5.3	−22.8, 12.3
Fish (g/day)	−1.0	−6.2, 4.3	−4.9	−13.2, 3.4
Red meat and poultry (g/day)	−3.3	−8.3, 1.8	−5.7	−15.3, 3.8
Processed meat (g/day)	0.2	−2.8, 3.3	4.9	−1.6, 11.3
Cheese (g/day)	0.4	−2.5, 3.3	1.4	−4.1, 7.0
Starches (pasta, rice, semolina, cereals, potatoes, tubers, bread) (g/day)	−2.7	−11.3, 6.0	−12.1	−29.3, 5.1
Whole-grain products (whole-grain cereals, bread, pasta, rice, flour) (g/day)	3.0	−2.0, 8.1	5.1	−4.4, 14.6
Fatty and sweet products (g/day)	**5.1** ^**d**^	**0.1, 10.2**	4.8	−4.9, 14.4
Sweet non-alcoholic beverages (g/day)	−0.8	−5.6, 4.1	3.2	−5.8, 12.2
Butter, margarine (g/day)	0.9	−0.6, 2.3	2.1	0.02, 4.2
Dairy products (cheese, milk, yogurt) (g/day)	−10.4	−21.6, 0.7	−12.2	−27.0, 2.5
Alcoholic beverages (g/day)	1.5	−7.4, 10.4	−17.3	−36.3, 1.3
Nutrients				
Total energy (kcal/day)	−17.9	−54.7, 18.8	4.1	−72.6 80.8
Proteins (g/day)	**−1.8** ^**d**^	**−3.2, −0.3**	−1.5	−3.6, 0.6
Total carbohydrates (g/day)	−1.0	−4.0, 2.0	0.8	−4.7, 6.2
Complex carbohydrates (g/day)	0.0	−2.4, 2.4	−0.8	−5.1, 3.5
Simple carbohydrates (g/day)	−1.0	−3.2, 1.2	1.8	−1.9, 5.4
Fibre (g/day)	−0.5	−1.1, 0.1	−0.1	−1.0, 0.7
Lipids (g/day)	1.3	−0.1, 2.6	1.7	−0.7, 4.0
Polyunsaturated fatty acids (g/day)	−0.1	−0.7, 0.4	−0.8	−1.8, 0.2
Monounsaturated fatty acids (g/day)	0.5	−0.2, 1.3	0.6	−0.7, 1.8
Saturated fatty acids (g/day)	**0.8** ^**d**^	**0.04, 1.6**	**1.6** ^**d**^	**0.3, 2.9**
Cholesterol (mg/day)	9.8	−3.4, 22.9	4.7	−17.5, 26.9
Omega 3 (g/day)	0.0	−0.1, 0.1	0.0	−0.2, 0.2
Omega 6 (g/day)	−0.1	−0.6, 0.4	−0.8	−1.7, 0.1
Calcium (mg/day)	−24.3	−48.9, 0.3	1.4	−40.7, 43.5
Magnesium (mg/day)	9.9	−0.5, 20.4	14.7	−0.6, 30.0
Sodium (g/day)	**323.2** ^**c**^	**245.5, 400.9**	**317.7** ^**c**^	**187.2, 448.2**
Beta carotene (μg/day)	−135.1	−423.9, 153.7	−111.8	−489.7, 266.1
Folate (μg/day)	**−18.2** ^**c**^	**−28.2 − 8.3**	-8.3	−26.3, 9.7
Vitamin C (mg/day)	**−10.5** ^**d**^	**−19.2, −1.2**	−0,1	−4.6, 5.0

*mPNNS-GS* modified French Programme National Nutrition Santé-Guideline Score. Use of bolded text in Table 3 highlights statistical significance

^a^Mixed models adjusted for total energy intake with random effects of the time and the period (before and after retirement)

^b^mPNNS-GS: adherence to nutritional guidelines score, based on 24 h dietary records, range 0–13.5

^c^ *P*-value <0.0001

^d^ *P*-value <0.01

#### Associations between transition to retirement and dietary intakes according to spousal retirement status and baseline household income

Significant interactions between spousal status to retirement and retirement were found regarding alcoholic beverages, total and complex carbohydrates, lipids and cholesterol in both sexes; in women, significant interactions were found regarding intakes of processed meat, simple carbohydrates, polyunsaturated fatty acids, omega 3, omega 6 and sodium while in men, interactions were significant for intakes of fruits, cheese, whole grain products, proteins, fibre and calcium.

Significant interactions between income at baseline and retirement were found regarding mPNNS score, fruits, and magnesium in both sexes. In women, significant interactions between income at baseline and retirement were found regarding folate, vitamin C and fibre while in men, interactions were significant for intakes of dairy products, alcoholic beverages, proteins, total carbohydrates, lipids, and saturated fatty acids.

In 436 (75.6% of the sample) individuals in a couple at baseline, some relationships between retirement and dietary intake were specific to spousal retirement status. Only in men for whom the spouse also retired, retirement was associated with a decrease in intake of alcoholic beverages and an increase in the intake of lipids (Additional file 1: Table S1). In analyses stratified by income at baseline, results showed that only in men belonging to the lowest income ﻿category (<1800 euros), a decrease in intake of dairy products and an increase in intake of lipids were observed with retirement (Additional file 2: Table S2).

## Discussion

The use of longitudinal repeated data in the years surrounding the retirement allowed a life course approach of the impact of retirement on dietary intakes and provided better understanding of associations between retirement, diet, demographic and socio-economic factors. Our study showed that, independently of changes over time, transition to retirement was associated with unhealthier dietary intakes such as decrease in adherence to nutritional guidelines and in intakes of fruits, proteins, and some vitamins as well as increase in intakes of fatty and sweet products, saturated fatty acids, and sodium. Changes of dietary intakes with retirement were particularly marked in men with the lowest income at baseline. In addition, differentiating individuals on spousal retirement highlighted some specific associations between dietary intakes and retirement.

To our knowledge, overall diet quality in relation to retirement has been studied using an index of adherence to recommendations in only one prospective study [13]. In contrast to our results, retirement was associated with an increase in healthy food habits. In a previous French study, the PNNS-GS score was positively associated with reduced risk of major chronic diseases, suggesting that a decrease in the score after retirement could have adverse consequences on health [33]. The decrease of overall diet quality after retirement could be explained by reduced spending on nutrient-dense foods, such as fruits which are more expensive, and an increased spending on more affordable products with higher contents of lipids or sodium [34]. Literature has shown that retirement is associated with a decrease in food expenditure [35] which may be due to the decrease in income associated with retirement. In France, the mean gross monthly retirement pension is 1216 euros, which represented 66% of the mean salary and 90% of the minimum salary in 2010 in France [36]. Changes in environmental conditions of meals could also impact dietary habits. Individuals stopping to have lunch at staff canteen or workplace, which are known to be associated with healthy eating habits might be another explanation to the decrease of the overall diet quality [37, 38]. After retirement individuals might perceive meal preparation as a stronger constraint, leading them to consume more convenience products which may have low nutritional quality and low costs. Then, meals may be less balanced than lunches at staff canteen, which have to follow national nutritional guidelines in France.

In our study, transition to retirement was also associated with a decrease in intakes of fruits and vitamins while previous studies have shown equivocal results [9, 16, 17]. In particular, the few prospective works which studied the impact of retirement on fruit consumption showed no significant association between retirement and fruit intake [9, 16]. Differences with previous studies could be explained by the fact that our study was based on repeated measures of dietary intake surrounding the retirement and not solely on data at baseline and at the end of the follow-up. As fruit intakes are associated with reduced risk of chronic diseases, decrease in their intake after retirement could have adverse consequences on health of retiring individuals [39]. Subsidies can increase intake of fruits and vegetables showing that cost of such products can be a barrier to consumption, particularly during this vulnerable period [40]. We observed a decrease in the intake of dairy products with retirement in men with low income. Dairy products could be protective against several chronic diseases [41]. A decrease in the intake of dairy products through retirement could potentially have negative health impact at an age where risk is higher [2].

In addition to the observed decrease in foods or nutrients beneficial to a healthy diet, an increase in components which intake should be limited such as lipids, saturated fatty acids, sodium, and fatty sweet products was also observed with retirement. High intake of saturated fatty acids, lipids, and sodium are associated with increased risk of cardiovascular diseases [42, 43]. These results were not concordant with a previous study, in which decrease in fat intake was observed after retirement [15]. Affordability and palatability of foods rich in fats and sodium or added fats with retirement may explain the observed increase in consumption. In stratified analyses, an increase in the intake of lipids was observed only in men with lowest income at baseline. This suggests that these individuals might be even more affected by the decrease in income due to retirement, leading them to buy more energy-dense and affordable products [34]. Particularly in men, income before retirement was related to specific changes in dietary intakes over retirement and seemed to be an important determinant of future food expenditure leading to decreased consumption of dairy products and increased consumption of affordable products.

Regarding changes in intakes according to spousal retirement, men whom spouse also retired during follow-up seemed to be particularly affected by retirement, leading these individuals to have unhealthier dietary intakes such as higher intakes of lipids. As women are often the main cook in French households [44], more available time after retirement may lead them to increase time devoted to cooking and consequently they may prepare more frequently traditional dishes -rich in fats - for the household [45]. In individuals whom spouses also retired, retirement could have an impact on both affordability and cooking practices. In addition, we observed a decrease in intake of alcoholic beverages after retirement in men whom spouse also retired during follow-up. Decrease in alcohol intake was not concordant with previous studies [12, 46]. When the retirement of the participant is concomitant with that of their spouse, the simultaneous decreases in both their incomes may act like a dual constraint on the household expenditures which might lead to reduced spending on non-essential foods such as alcoholic beverages. In addition, it has been shown that retirement of one individual and of his/her spouse reduced the individual’s spending on eating out. Social occasions where alcohol is consumed, such as going to a restaurant, may be less frequent after retirement, leading to a decreased alcohol intake in men.

### Strengths and limitations

Since the NutriNet-Santé cohort includes volunteers, more subjects were women, belonged to high education group and had a healthier lifestyle than the general population [47], and were probably more interested in nutrition than the general population. In particular, the overrepresentation of women in our sample could be explained by the fact that women are more likely to participate in voluntary-based health and epidemiological studies, whatever the field concerned [48]. Thus, caution is needed when interpreting and generalizing results. Changes in dietary intake with retirement might be probably larger in the general population. Regarding estimations of dietary intakes, studies investigating the validity of our web-based, self-reported dietary record tool against biomarkers showed that our tool performs well in estimating several nutrients and food intakes [24, 25]. Strength of our study was its reliance on repeated three non-consecutive-day dietary records and repeated socioeconomic data in the years surrounding the retirement (one to three before or after retirement). Further analyses according to baseline income and spousal retirement were also strength, allowing us to further explore the associations and to interpret more accurately the impact of retirement on dietary intakes. However, the relative small size of our sample may have reduced the statistical power in stratified analyses. Length of period between the retirement and the end of the follow-up was not as accurate as the exact real duration. Indeed, we used the year of the completion of the socio-demographic questionnaire as a proxy for the date of retirement. This may have reduced the ability to differentiate specific changes due to retirement such as short-term or long-term effects. Another strength of our analyses is that we performed comparisons within subject of changes in intakes using the binary period variable (before/after retirement) as indicator of the transition to retirement. This method allowed us to assess accurately the associations between transition to retirement and changes in dietary intakes. Although health events such as occurrence of chronic diseases or cancer are likely to appear later in life and to modify employment status and dietary habits, they were not included in our models. A combined approach might be of interest to clarify the relative roles of each determinant. Caution is also needed when generalizing to other countries because important variation in policies for healthcare and retirement exists and could explain differences between studies.

## Conclusions

Transition to retirement was associated with unhealthier dietary intakes, such a decrease in the overall diet quality and intakes of recommended foods and nutrients and an increase in nutrients or foods which consumption should be limited. Changes in dietary intakes appear to be particularly marked in men with low income at baseline. Identifying vulnerable periods in the life course, such as retirement, could lead to improve the implementation of nutritional interventions in at risk populations. For instance, targeted interventions before age of retirement on methods to optimize diet quality with reduced budget could be of interest. Further investigations on the associations between retirement, food budget and diet quality but also more precise knowledge on the mediating effect of diet between retirement and health would be useful to public health strategies.

## Additional files

Additional file 1: Table S1. Associations between changes in dietary intakes and retirement according to spousal retirement status in men and women (NutriNet-Santé Study). (DOCX 20 kb)

Additional file 2: Table S2: Associations between changes in dietary intakes and transition to retirement according to baseline income in women and men (NutriNet-Santé Study). (DOCX 20 kb)

## Abbreviations

HU: Household unit
mPNNS GS: Modified French Programme National Nutrition Santé - Guideline Score
PNNS GS: French Programme National Nutrition Santé - Guideline Score
