# Supplementary material for: Associations between transition to retirement and changes in dietary intakes in French adults (NutriNet-Santé cohort study)
Source: Int J Behav Nutr Phys Act. 2017 May 30;14:71. doi: 10.1186/s12966-017-0527-6 (PMC5450356; doi:10.1186/s12966-017-0527-6)
Supplement: Supplementary file 1 — Associations between changes in dietary intakes and retirement according to spousal retirement status in men and women (NutriNet-Santé Study). (DOCX 20 kb) [file 12966_2017_527_MOESM1_ESM.docx]

Additional file 1 Table S1.

Associations between changes in dietary intakes and retirement according to spousal retirement status in men and women (NutriNet-Santé Study)^a^

|  | **Women** | | | | **Men** | | | |
| --- | --- | --- | --- | --- | --- | --- | --- | --- |
|  | **Individuals whom spouse did not retire during follow-up (N=186)** | | **Individuals whom spouse retired during follow-up (N=101)** | | **Individuals whom spouse did not retire during follow-up (N=95)** | | **Individuals whom spouse retired during follow-up (N=54)** | |
|  | **Beta** | **99% Confidence Interval** | **Beta** | **99% Confidence Interval** | **Beta** | **99% Confidence Interval** | **Beta** | **99% Confidence Interval** |
| **Food groups** |  |  |  |  |  |  |  |  |
| Fruit (g/day) |  |  |  |  | 16.7 | -21.7, 55.2 | -1.9 | -59.3, 55.6 |
| Processed meat (g/day) | 1.0 | -3.8, 5.9 | 1.3 | -4.4, 7.1 |  |  |  |  |
| Cheese (g/day) |  |  |  |  | 2.7 | -4.9, 10.3 | 1.1 | -10.1, 12.3 |
| Whole-grain products (whole-grain cereals, bread, pasta, rice, flour) (g/day) |  |  |  |  | 7.9 | -6.2, 22.0 | -4.2 | -14.6; 6.2 |
| Alcoholic beverages (g/day) | -1.7 | -13.8, 10.4 | -8.9 | -24.4, 6.6 | -9.3 | -37.1, 18.4 | **-33.9^e^** | **-62.8, -5.0** |
| **Nutrients** |  |  |  |  |  |  |  |  |
| Proteins (g/day) |  |  |  |  | -1.0 | -3.8, 1.7 | -1.4 | -5.6, 2.9 |
| Total carbohydrates (g/day) | -1.5 | -5.8, 2.9 | 3.3 | -2.9, 9.5 | 3.6 | -4.6, 11.7 | -4.4 | -12.2, 3.4 |
| Complex carbohydrates (g/day) | -0.6 | -4.0, 2.8 | 2.5 | -2.7, 7.6 | 0.1 | -6.5, 6.7 | -1.6 | -8.1, 5.0 |
| Simple carbohydrates (g/day) | -0.7 | -3.8, 2.4 | 0.8 | -4.1, 5.6 |  |  |  |  |
| Fibre (g/day) |  |  |  |  | 0.5 | -0.8, 1.7 | -1.2 | -2.6, 0.2 |
| Lipids (g/day) | 1.4 | -0.7, 3.4 | 0.2 | -2.5, 2.8 | 0.2 | -3.4, 3.8 | **4.0^e^** | **0.3, 7.8** |
| Polyunsaturated fatty acids (g/day) | -0.1 | -0.9, 0.7 | -0.8 | -1.9, 0.4 |  |  |  |  |
| Cholesterol (mg/day) | 13.7 | -4.8, 32.1 | 2.5 | -26.7, 31.7 | 5.5 | -24.4, 35.4 | 2.8 | -43.6, 49.3 |
| Omega 3 (g/day) | 0.0 | -0.1, 0.1 | -0.1 | -0.3, 0.1 |  |  |  |  |
| Omega 6 (g/day) | -0.1 | -0.8, 0.6 | -0.6 | -1.6, 0.4 |  |  |  |  |
| Calcium (mg/day) |  |  |  |  | 14.4 | -44.9, 73.7 | -15.5 | -93.0, 62.0 |
| Sodium (g/day) | **333.5^c^** | **208.5, 458.4** | **362.5^c^** | **235.4, 489.7** |  |  |  |  |
| \| Abbreviations: mPNNS-GS : modified French Programme National Nutrition Santé-Guideline Score. Use of bolded text in Table S1 highlights statistical significance.  a Mixed models adjusted for total energy intake with random effects of the time and the period (before and after retirement)  b mPNNS-GS: adherence to nutritional guidelines score, based on 24h dietary records, range 0-13.5  c P-value < 0.0001 \| \| --- \| | | | | | | | | |
