# Supplementary material for: Associations between transition to retirement and changes in dietary intakes in French adults (NutriNet-Santé cohort study)
Source: Int J Behav Nutr Phys Act. 2017 May 30;14:71. doi: 10.1186/s12966-017-0527-6 (PMC5450356; doi:10.1186/s12966-017-0527-6)
Supplement: Supplementary file 2 — Associations between changes in dietary intakes and transition to retirement according to baseline income in women and men (NutriNet-Santé Study). (DOCX 20 kb) [file 12966_2017_527_MOESM2_ESM.docx]

Additional file 2 Table S2: Associations between changes in dietary intakes and transition to retirement according to baseline income in women and men^a^

|  | **Women** | | | | | | **Men** | | | | | |
| --- | --- | --- | --- | --- | --- | --- | --- | --- | --- | --- | --- | --- |
|  | **<1,800 euros**  **(N=63)** | | **1,800-2,700 euros**  **(N=108)** | | **>2,700 euros**  **(N=199)** | | **<1,800 euros**  **(N=30)** | | **1,800-2,700 euros**  **(N=29)** | | **>2,700 euros**  **(N=108)** | |
|  | **Beta** | **99% Confidence Interval** | **Beta** | **99% Confidence Interval** | **Beta** | **99% Confidence Interval** | **Beta** | **95% Confidence Interval** | **Beta** | **95% Confidence Interval** | **Beta** | **95% Confidence Interval** |
| Adherence to nutritional recommendations (mPNNS-GS) ^b^ (range 0-13.5) | **-0.6^e^** | **-1.2, -0.1** | **-0.5^d^** | **-0.8, -0.2** | **-0.4^c^** | **-0.6, -0.1** | -0.3 | -1.0, 0.4 | -0.3 | -0.9, 0.4 | -0.1 | -0.4, 0.2 |
| **Food groups** |  |  |  |  |  |  |  |  |  |  |  |  |
| Fruit (g/day) | -13.8 | -58.9, 31.4 | -29.3 | -61.5, 2.9 | -12.4 | -35.9, 11.2 | 23.7 | -29.1, 86.6 | 3.4 | -61.1, 67.9 | 18.2 | -20.7, 57.1 |
| Dairy products (cheese, milk, yogurt) (g/day) |  |  |  |  |  |  | **-36.5^e^** | **-72.1, -0.9** | -12.0 | -45.5, 21.4 | -6.6 | -25.6, 12.4 |
| Alcoholic beverages (g/day) |  |  |  |  |  |  | -45.7 | -103.4, 12.1 | 26.5 | -27.2, 80.3 | -20.2 | -42.0, 1.6 |
| **Nutrients** |  |  |  |  |  |  |  |  |  |  |  |  |
| Proteins (g/day) |  |  |  |  |  |  | -4.9 | -10.0, 0.2 | -3.2 | -8.3, 1.8 | -0.2 | -4.0, 0.7 |
| Total carbohydrates (g/day) |  |  |  |  |  |  | -5.4 | -20.3, 9.6 | 3.7 | -8.2, 15.5 | 1.1 | -6.2, 8.4 |
| Fibre (g/day) | -0.7 | -2.1, 0.7 | -0.6 | -1.8, 0.5 | -0.4 | -1.1, 0.4 |  |  |  |  |  |  |
| Lipids (g/day) |  |  |  |  |  |  | **7.1^e^** | **0.8, 13.4** | 0.0 | -6.9, 6.9 | 0.9 | -2.1, 3.9 |
| Saturated fatty acids (g/day) |  |  |  |  |  |  | 4.0 | -0.1, 8.0 | 1.1 | -1.9, 4.1 | 1.2 | -0.4, 2.8 |
| Magnesium (mg/day) | 5.4 | -22.3, 33.1 | 6.3 | -14.2, 26.9 | **15.2^e^** | **0.7, 29.7** | 32.9 | -10.8, 76.6 | 20.5 | -25.9, 66.9 | 7.7 | -10.9, 26.2 |
| Folate (µg/day) | -6.2 | -28.8, 16.4 | **-20.7^e^** | **-40.4, -0.9** | **-22.0^d^** | **-36.7, -7.2** |  |  |  |  |  |  |
| Vitamin C (mg/day) | -2.0 | -16.9, 13.0 | -11.1 | -23.9, 1.7 | -15.4 | -30.8, 0.1 |  |  |  |  |  |  |
| Abbreviations: mPNNS-GS : modified French Programme National Nutrition Santé-Guideline Score. Use of bolded text in Table S2 highlights statistical significance. | | | | | | | | | | | | |
| a Mixed models adjusted for total energy intake with random effects of the time and the period (before and after retirement) | | | | | | | | | | | | |
| b mPNNS-GS: adherence to nutritional guidelines score, based on 24h dietary records, range 0-13.5 | | | | | | | | | | | | |
| c P-value < 0.0001 | | | | | | | | | | | | |
| d P-value < 0.001 | | | | | | | | | | | | |
| e P-value < 0.01 | | | | | | | | | | | | |
